# Isolated central nervous system relapses in patients with high-risk neuroblastoma -clinical presentation and prognosis: experience of the Polish Paediatric Solid Tumours Study Group

**DOI:** 10.1186/s12885-022-09776-x

**Published:** 2022-06-25

**Authors:** Aleksandra Wieczorek, Joanna Stefanowicz, Marcin Hennig, Elzbieta Adamkiewicz-Drozynska, Marzena Stypinska, Bozenna Dembowska-Baginska, Zuzanna Gamrot, Mariola Woszczyk, Julia Geisler, Tomasz Szczepanski, Szymon Skoczen, Marek Ussowicz, Monika Pogorzala, Szymon Janczar, Walentyna Balwierz

**Affiliations:** 1 Department of Paediatric Oncology and Haematology, Faculty of Medicine, Jagiellonian University, Medical College, Krakow, Poland; 2 Department of Paediatrics, Haematology and Oncology, Medical University of Gdansk, Gdansk, Poland; 3 Department of Oncology, The Children Memorial Health Institute in Warsaw, Warsaw, Poland; 4 Unit of Paediatric Haematology and Oncology, City Hospital, Chorzow, Poland; 5 Department of Paediatric Haematology and Oncology, Medical University of Silesia, Zabrze, Poland; 6 Department and Clinic of Paediatric Oncology, Haematology and Bone Marrow Transplantation, Wroclaw Medical University, Wroclaw, Poland; 7 Paediatric Haematology and Oncology, Collegium Medicum, Nicolaus Copernicus University, Bydgoszcz, Poland; 8 Department of Paediatrics, Oncology and Haematology, Medical University of Lodz, Lodz, Poland

**Keywords:** Central nervous system, Clinical course, Isolated relapse, Metastases, Neuroblastoma

## Abstract

Although isolated central nervous system (CNS) relapses are rare, they may become a serious clinical problem in intensively treated patients with high-risk neuroblastoma (NBL). The aim of this study is the presentation and assessment of the incidence and clinical course of isolated CNS relapses. Retrospective analysis involved 848 NBL patients treated from 2001 to 2019 at 8 centres of the Polish Paediatric Solid Tumours Study Group (PPSTSG). Group characteristics at diagnosis, treatment and patterns of relapse were analysed. Observation was completed in December 2020. We analysed 286 high risk patients, including 16 infants. Isolated CNS relapse, defined as the presence of a tumour in brain parenchyma or leptomeningeal involvement, was found in 13 patients (4.5%; 8.4% of all relapses), all of whom were stage 4 at diagnosis. Isolated CNS relapses seem to be more common in young patients with stage 4 MYCN amplified NBL, and in this group they may occur early during first line therapy. The only or the first symptom may be bleeding into the CNS, especially in younger children, even without a clear relapse picture on imaging, or the relapse may be clinically asymptomatic and found during routine screening. Although the incidence of isolated CNS relapses is not statistically significantly higher in patients after immunotherapy, their occurrence should be carefully monitored, especially in intensively treated infants, with potential disruption of the brain-blood barrier.

## Background

Neuroblastoma (NBL) is the most common extracranial solid tumour in children and one of the most common causes of death in paediatric oncology [1, 2]. The treatment results improved after implementation of new treatment modalities such as high-dose chemotherapy (HDC) followed by autologous stem cell rescue (ASCR), retinoid therapy and immunotherapy, including patients in the high-risk group [3–6]. However, concerns have been raised that intensive treatment may modify the pattern of relapses.

Central nervous system (CNS) involvement at diagnosis and isolated CNS relapses are rare in children with primary extracranial tumours, including NBL. There are limited data presenting brain metastases in children, but clinical reports on different extracranial tumours suggest a frequency of 1.5–4.9% [7–9], with frequency which is even higher in autopsy studies (6–13%) [10, 11]. The estimated 3-year risk of CNS relapses in NBL reaches 8% [12], with CNS being either isolated or one of the sites involved in multiorgan relapse.

The CNS can act as a “sanctuary site” for cancer cells because the blood-brain barrier may impede the penetration of most chemotherapeutic agents [13]. Additionally, antibodies used in immunotherapy do not penetrate into the brain [14], which may influence the frequency of isolated CNS relapses in children undergoing immunotherapy.

The aim of the study is the presentation, assessment of incidence, and assessment of the clinical course of isolated CNS relapses in NBL patients treated at 8 centres of the Polish Paediatric Solid Tumours Study Group (PPSTSG).

## Patients and methods

From 1^st^ January, 2001 to 31^st^ December, 2017, 848 patients with NBL were diagnosed at 8 centres of the PPSTSG, including 286 patients with high risk disease. The data were collected till December 2019 and the follow-up was completed in December 2020. The high-risk group included 260 stage 4 patients (including 9 infants with MYCN amplification and 251 patients over 1 year of age, irrespective of MYCN status), 23 patients with stage 2 or 3 with MYCN amplification (including 4 infants), and 3 infants with 4 s MYCN amplified tumours. The mean age at diagnosis was 43.4 months (range 0.2 – 186.3). The characteristics of the group are presented in Table 1.

**Table 1:** Characteristics of high-risk neuroblastoma patients

Number of patients	848
Number of high-risk patients	286
Stage 4 patients > 1 year of age	251
Stage 4 infants with MYCN amplification	9
Patients with localised disease with MYCN amplification > 1 year of age	19
Infants with localised MYCN amplified tumours	4
Stage 4 s with MYCN amplification	3
MYCN status (n)	
Amplification present	116
No amplification	119
Unknown	51
Age (months)	
range	0.8 – 186.3
mean	43.3
median	32.4

For the high-risk group, children were treated according to HR-NBL-1/SIOPEN (High Risk Neuroblastoma Study 1.0 of SIOP-Europe) protocols [15, 16], including intensive chemotherapy induction (Rapid COJEC – alternating cycles of vincristine, carboplatin and etoposide; vincristine and cisplatin; and vincristine, etoposide and cyclophosphamide; in a case of poor response, 2 cycles of topotecan, vincristine and doxorubicin could have been added) and HDC (busulfan, melphalan or carboplatin, etoposide and melphalan) with ASCR. The details of treatment are reported elsewhere [15, 16]. Surgery, radiotherapy of the primary tumour site (21 Gy) and 13-cis retinoic acid (160 mg/m^2^/day for 14 days, 6 cycles every 4 weeks) were used as a standard in all patients. From 2012, children also received immunotherapy (ch14.18cho, 100 mg/m^2^/cycle, 5 cycles). The other protocols used included prolonged chemotherapy mostly according to the Japanese protocol [17] (vincristine, cisplatin, doxorubicin, cyclophosphamide, no HDC) or other individually established chemotherapy protocols, surgery and radiotherapy of the primary tumour site in most cases (dose 21–36.5 Gy). Therapy with 13-cis retinoic acid was introduced depending on clinicians’ decisions.

Altogether, 205 children received therapy according to the HR-NBL-1/SIOPEN protocol (the first group), and 81 patients had different chemotherapy protocols, without HDC (the second group). In 91 children out of 205 (44.4%) treated according to HR-NBL-1/SIOPEN, immunotherapy with ch14.18cho was given in the first line treatment.

**Fig. 1:**
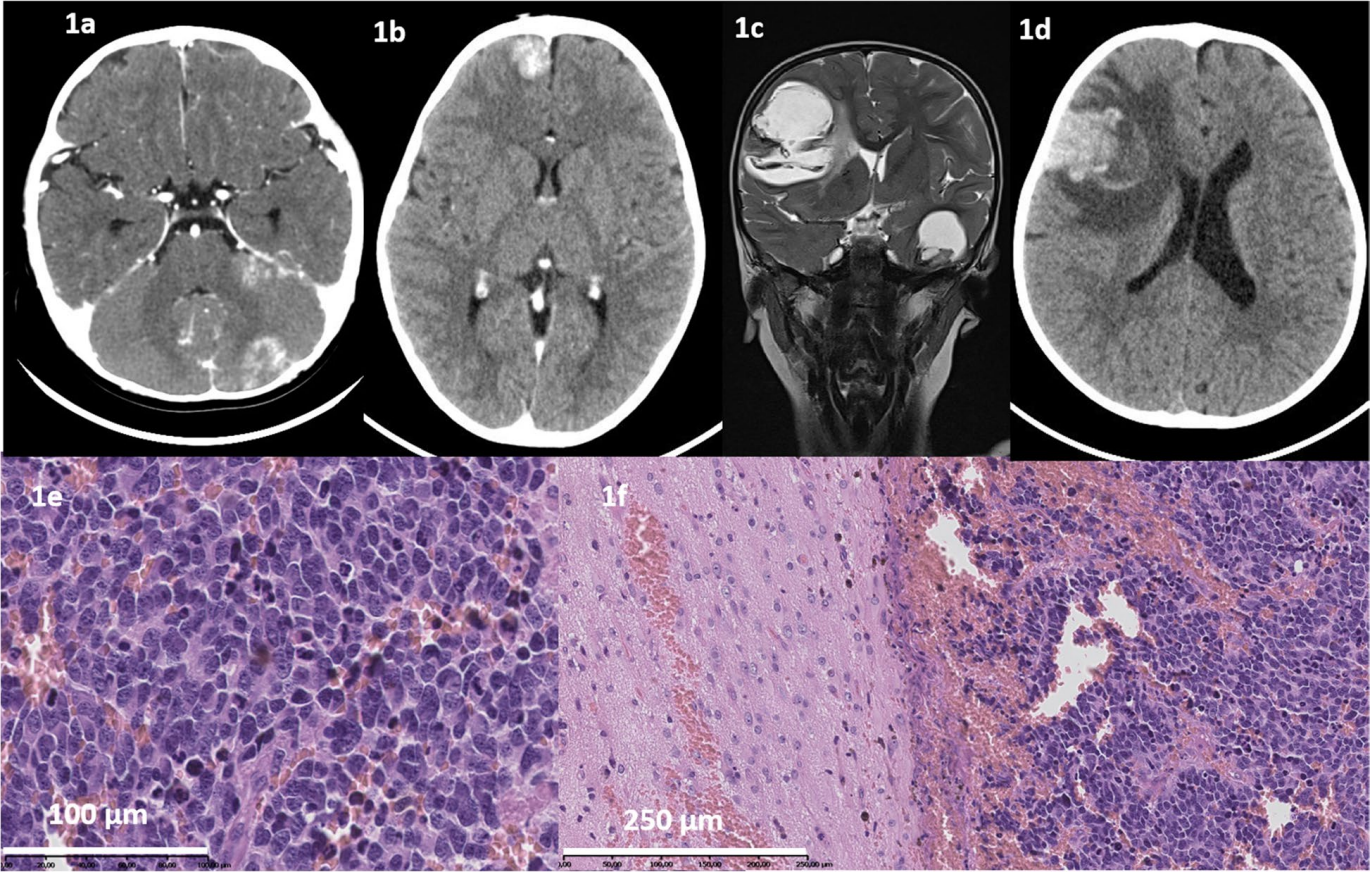
**a**. Computed tomography (CT) of the head, routine examination after induction. Tumour at area of tent of cerebellum, 24 × 14 mm, with possible bleeding to tumour, irregular masses on both sides of tent of cerebellum and in interpedicular cistern. **b**. In the right frontal lobe, the focal lesion 19 × 19 mm with small calcifications. **c**. Magnetic resonance (MRI), metastases in both hemispheres, left frontal lobe 46 × 61 mm, left temporal lobe 36 × 27 mm, bleeding to tumour. **d**. At the right frontal lobe the focal lesion 38 × 35x36mm, with oedema; mass effect – shift of the middle line structures to the left **e**.) NBL undifferentiated subtype, removed CNS tumor. Hematoxylin–eosin staining, 45 × **f**. NBL undifferentiated subtype, in removed CNS tumor – on the right site.. Hematoxylin–eosin staining, 20x

Isolated CNS relapse was defined as parenchymal (Fig. 1a-d) or leptomeningeal involvement, with or without the presence of NBL cells in the cerebrospinal fluid, and/or NBL cells found on histopathological examination of brain tumour or haematoma (Fig. 1e-f). Patients with CNS metastases originating in the skull bones were excluded from the analysis. The relapse was defined as isolated if the CNS was the only site of relapse in patients in complete remission or the relapse was the only new lesion in patients with stable disease at any other sites. All patients had either full evaluation of disease status (imaging, bone marrow examination and I^123^- or I^131^- metaiodobenzylguanidine (MIBG) scintigraphy) or *post mortem* examination to exclude other potential metastatic lesions. Only the first relapses were analysed.

The study was carried out in accordance with the Declaration of Helsinki and the recommendations of the Ethics Committee of Jagiellonian University Medical College, Kraków, Poland. All treatment protocols were approved by the Ethics Committee of the Jagiellonian University Medical College and legal representatives signed the informed consent for treatment and data analysis. No additional consent is required for retrospective analysis. Statistical analysis was performed with Statistica Software; the chi-square test and t-test were used, with a significance level 0.05.

## Results

Among 286 children with diagnosed HR-NBL, relapse was diagnosed in 161 (56.3%), including 103 (50.2%) relapses in 205 children treated with the SIOPEN protocol and 59 (72.8%) relapses in 81 children treated with conventional chemotherapy. The difference between these 2 groups was statistically significant (*p* = 0.0001). From 2001 to 2019, isolated CNS relapses were confirmed in 13 patients (8.4% of all relapses). Incidence of isolated CNS relapses was 10.7% in the first group (11 out of 103 relapses) and 3.4% in the second group (2 out of 59 relapses). The difference between groups was not statistically significant (*p* = 0.1).

Taking into consideration the employment of immunotherapy, 28 (30.8%) relapses occurred in 91 patients treated with immunotherapy and in 118 (63.1%) of 195 patients treated without immunotherapy in the first line treatment. The difference between groups was statistically significant (*p* < 0.0001). Six (6.6%) cases of isolated CNS relapse were diagnosed among 91 patients in whom immunotherapy was employed in the first line therapy and 7 cases (3.6%) among 195 patients without immunotherapy in the first line treatment. The difference between the groups was not statistically significant (*p* = 0.26). In the group treated with SIOPEN protocols, the CNS relapse occurred before the time of scheduled immunotherapy in five patients, during immunotherapy in three patients, and after immunotherapy (3–6 months) in three patients.

Clinical data on patients with isolated CNS relapse are presented in Tables 2 and 3.

**Table 2:** Patients with isolated relapse in CNS group characteristics at the time of the first diagnosis

Pts	Age at Dx (y, m)	Gender	Stage at Dx	Primary site Metastases at diagnosis	MYCN amplification/ Type of pathology	Skull bones involvement at Dx	First line therapy
1	2y 2 m	F	4	Adrenal gland Bones, BM, LN	Yes UH	Yes	HR-NBL SIOPEN No anti-GD2
2	2y 2 m	M	4	Retroperitoneal space Bones, BM, LN	Yes UH	Yes – intracranial penetration	HR-NBL SIOPEN No anti-GD2
3	5 m	F	4	Adrenal gland, Bones, BM, liver, kidneys, lungs, chest wall	Yes UH	No	HR-NBL SIOPEN No anti-GD2
4	3 m	F	4	Adrenal gland Bones, BM, LN, lungs, liver, kidneys	Yes UH	No	HR-NBL SIOPEN With anti-GD2
5	2y 6 m	F	4	Adrenal gland Bones, BM	Not evaluated UH	Yes	Conventional chemotherapy
6	3y 7 m	F	4	Mediastinum Bones, BM, LN, CSN, pleura	No UH	Yes – CNS infiltration	HR-NBL SIOPEN with anti-GD2
7	2y 7 m	M	4	Adrenal gland Bones, BM, LN	No UH	Yes	Conventional chemotherapy
8	10 m	M	4	Retroperitoneal space BM	Yes UH	No	HR-NBL SIOPEN No anti-GD2
9	11 m	M	4	Adrenal gland Bones, BM, LN	Yes UH	Yes – infiltration of skull base	HR-NBL SIOPEN With anti-GD2
10	2y 4 m	F	4	Retroperitoneal space Bones, BM	No FH	Yes	HR-NBL SIOPEN No anti-GD2
11	1y 8 m	M	4	Adrenal gland LN, mediastinum, bones, BM	Yes UH	Yes	HR-NBL SIOPEN With anti-GD2
12	1y 11 m	M	4	Adrenal gland LN, bones, BM	Yes UH	Yes – penetrating to CNS	HR-NBL SIOPEN With anti-GD2
13	6y 1m2	M	4	Retroperitoneal space Bones, BM	No UH	No	HR-NBL SIOPEN With anti-GD2

*Dx* diagnosis, *y* year, *m* month, *M* male, *F* female, *UH* unfavourable histopathology, *FH* favourable histopathology, *BM* bone marrow, *LN* lymph nodes

**Table 3:** CNS relapse presentation, treatment, and course of disease

Pt	Time from diagnosis to CNS relapse (months)	Relapse presentation	Relapse therapy	Disease status and time to relapse	Disease course after relapse
1	5	Facial nerve palsy, strabismus Tumour in CT NBL cells in CSF and in removed tumour	Surgery, RTX, chemotherapy, intrathecal chemotherapy	CR; on treatment after induction and surgery	Local progression 6 months from relapse, death 7 months from relapse
2	6	Intracranial bleeding No tumour in CT NBL cells in removed tumour	Surgery	CR; on treatment – before HDC	Died 2 weeks after event
3	5	Accidental diagnosis CT: infiltration of meninges, bleeding into the tumour	Chemotherapy, intrathecal chemotherapy	PR; on treatment - after induction	Died 3 weeks after event bleeding into tumour
4	12	Intracranial bleeding No radiological symptoms NBL in *postmortem* examination	No therapy	CR; on treatment - therapy of MRD	Died 5 days after event
5	8	Headache, nausea, vomiting Tumour in CT NBL cells in removed tumour	Surgery, radiotherapy, chemotherapy, 13 cis RA	CR; after end of treatment	CR in CNS; disseminated bone relapse 39 months after relapse; no 3rd line treatment. Died of disease
6	15	Facial nerve palsy	Chemotherapy	PR; on treatment – therapy of MRD	Local progression and death 3 months after relapse
7	26	Headache, nausea, vomiting Tumour in CT	Chemotherapy, radiotherapy, no surgery (tumour regression on chemotherapy)	CR; after end of treatment	Alive in CR > 9 years after relapse
8	8	Intracranial bleeding No radiological symptoms NBL in *postmortem* examination	No therapy	CR; on treatment – after radiotherapy	Died 2 days after event
9	20	Headache, nausea, vomiting 2 tumours in CT NBL cells in removed tumour	Surgery, chemotherapy, radiotherapy, ibritumomab	CR; 3 months after end of treatment	Alive in CR, 2,5 years after relapse
10	8	Intracranial bleeding No radiological symptoms NBL in *postmortem* examination	No therapy	PR; on treatment – after HDC, before ASCR	Died 7 days after event
11	16	Headache 3 metastases in CT	No therapy	CR, during immunotherapy	Died of disease 3 months after event
12	14	No symptoms; mild headache on a day of planned CT Tumour on CT – routine screening	Surgery, chemotherapy, radiotherapy	CR: during immunotherapy	Alive, 2^nd^ CNS relapse after 9 months
13	20	Headache, vomiting Infiltration of meninges in MRI, NBL cells in cerebrospinal fluid	Chemotherapy, radiotherapy	CR; 6 months after end of immunotherapy	Died of disease progression

*Dx* diagnosis, *NBL* neuroblastoma, *CNS* central nervous system, *CT* computed tomography, *RTX* radiotherapy, *CR* complete remission, *PR* partial remission, *MRD* minimal residual disease, *MRI* magnetic resonance imaging

### Clinical and biological data on children with isolated CNS relapse

In 12 out of 13 patients, MYCN status is known. Eight (66.7%) out of twelve patients had MYCN amplification. All patients had unfavourable histopathology at diagnosis. Although all patients were stage 4 at the time of diagnosis, the metastatic pattern was not constant. Nine (69.2%) out of 13 patients had skull bone involvement and in 3 patients (23.1%), the CNS was primarily involved by continuity with skull bone infiltration.

Pathologic verification of CNS lesions at relapse was obtained in 11 patients, including four with NBL cells found in haematomas, three with NBL cells presented in cerebrospinal fluid and five with histopathological evaluation of tissue received after tumour removal (one of them had also previous positive CSF evaluation).

### Characteristics of CNS relapse

The time from the diagnosis to relapse was 5–26 months (mean 12.5 months, median 12.0 months).

The time to relapse was much shorter in young children with intracranial bleeding being the first presentation of relapse or accidental relapse diagnosis (5–12 months, mean 7.2) than in children with symptomatic relapse (8–26 months, mean 17.4). The difference is statistically significant (*p* = 0.0002). 

Eight relapses were diagnosed in 16 infants with MYCN amplification, seven in stage 4 and one in stage 3 patients. Four of them (50%), all in stage 4 infants, were isolated CNS relapses. In three infants with isolated CNS relapse, ASCR was not carried out because of disease progression that occurred during intensive induction chemotherapy or just before planned HDC.

### Clinical manifestation of isolated central nervous system relapse

In intensively treated patients, especially the youngest ones, isolated CNS involvement may be asymptomatic or rapid symptoms of intracranial bleeding may occur, without previous symptoms of CNS involvement.

Intracranial bleeding was the first clinical symptom in 4 (30.2%) out of 13 patients, including three infants, all with MYCN amplification, with acute symptoms of increased intracranial pressure and early invagination. Two of them were diagnosed before planned ASCT and 2 either just before or during immunotherapy. Imaging (computed tomography (CT) and/or magnetic resonance imaging (MRI)) done in three children revealed no evident tumour mass, only intracranial bleeding, and in 1 patient there was a suspicion of bleeding into the tumour. Assessment of meninges on imaging was difficult as no contrast was given. The presence of disseminated neuroblastoma cells in haematoma together with infiltration of meninges with NBL cells was revealed during histopathological examinations in all patients. One patient had a surgery at the time of symptom occurrence, and NBL infiltration was found during *post mortem* examination in three patients, as rapid progression of invagination and brain death occurred. In all these patients, the symptoms progressed very rapidly. Two of them were hospitalised at the time of episode, the other one, with no symptoms, was seen in an outpatient clinic on the day of the episode. Only one out of four had a mild headache a few days before the episode which was interpreted to be caused by teething. All but one patient was younger than 18 months at the time of relapse, so the potential symptoms were difficult to assess. No CNS lesions were visible in MIBG imaging or/and in CT/MRI done at any time before the relapse.

In two out of four patients, there was an episode of mild head trauma on the day of bleeding, such as falling down while walking (13 and 15 months old at the time of relapse). In both cases, the accident happened in the presence of parents with no loss of consciousness or any other symptoms directly after the accident.

In 2 out of 13 patients (15.4%), there were no clinical symptoms of CNS involvement and the relapse was diagnosed during routine examinations after induction chemotherapy (stage 4 infant with MYCN amplification, leptomeningeal involvement (Fig. 1a) and after 2 cycles of anti-GD2 (stage 4 infant with MYCN amplification at diagnosis, only mild headache on the day of planned CT). The first patient, in spite of the chemotherapy provided, had bleeding to the tumour and died in the course of this episode three weeks after CNS relapse. In the second one, a complete remission was obtained after surgery and chemotherapy, but the second isolated CNS relapse occurred during treatment at the time of the last evaluation.

The other 7 (53.8%) children presented with neurological symptoms of increased intracranial pressure (nausea, vomiting, headache) or focal symptoms, mainly disturbances of cranial nerves (strabismus, paresis of the facial nerve). Symptoms lasted for a few days before the diagnosis and were the reason for performing CNS imaging. All of them were over 18 months old at the time of diagnosis and the time to progression was longer (8–26 months, mean 17.4 months from the first diagnosis).

Only in 3 out of 13 patients the time to relapse was 20 months or longer. including both patients who survived and who are disease free. One of them is alive without the disease > 5 years from the relapse diagnosis and the second one 2.5 years from the relapse diagnosis. The first patient received prolonged chemotherapy as the first line treatment, without HDC, and CNS relapse was treated with chemotherapy according to a brain tumour protocol and radiotherapy without surgery as the tumour regressed after treatment. The second one received the HR-NBL-1/SIOPEN protocol with immunotherapy as the first line treatment, and CNS relapse was treated with surgery, chemotherapy, RTX, and intraventricular compartmental radioimmunotherapy.

## Discussion

There are few data reported in the literature on the incidence of isolated CNS relapses in patients with high-risk NBL. The previously reported rate of incidence of CNS relapses was from 2.3% [18] to 16% [19] and up to 25% in small institution studies [20], with a mean of 3.8% [12]. However, most studies did not report only isolated CNS relapses. It is estimated that these account for about 50% of all CNS relapses [12, 18]. In the German Childhood Cancer Registry, 85 patients with CNS involvement were identified, including 57 with isolated CNS relapse [21].

Among 127 patients with stage 4 NBL over one year of age diagnosed at Memorial-Sloan-Kettering, eight patients (6%) developed CNS relapses. In this cohort, there was a tendency towards a higher number of CNS relapses among patients treated with immunotherapy without HDC than in patients that received HDC without immunotherapy (7 out of 67 vs 1 out of 60, respectively) [22]. The retrospective analysis performed by the Children’s Oncology Group showed CNS relapses at first recurrence in 8 out of 434 patients (2%) [18], and in the French cohort, CNS relapses were diagnosed in 8 out of 127 patients (6%) [12]. In the German study, the incidence of CNS relapses was reported in 49 out of 451 patients (11%) treated with HDC [23]. In the Chinese series, brain metastases occurred in 11 out of 106 patients (10.4%), accounting for 20% of all relapses [24]. The frequency of CNS relapses seems not to change over time (1985–2000 [12], 1990–2010 [21]). However, the influence of immunotherapy is still unclear. There are data supporting the thesis that by reducing the incidence of systemic relapses, the number of isolated CNS relapses increases [25], but in the SIOPEN analysis the increased risk of CNS relapse after immunotherapy was not confirmed [26]. In our study, although the incidence of isolated CNS relapses was higher in intensively treated patients and after immunotherapy, the difference was not statistically significant.

The studies referred to above, except for Matthay et al. [12], reported on data from studies including children over one year of age at the time of diagnosis. As a relatively high number of infants were observed in our cohort, and in this group isolated CNS relapses were 50% of all relapses, the number of CNS relapses may be underestimated in the whole high-risk group, also including patients under one year of age with MYCN amplification. Additionally, in some studies, only patients who received HDC were assessed [23], which excluded early relapses, which seem to be more common in young intensively treated patients.

In the current study, we presented 13 patients with isolated CNS neuroblastoma relapse. The pattern of relapses seems to change with treatment intensification, resulting in early relapses, taking the form of intracranial bleeding, especially in infants.

There are no clear risk factors for isolated CNS relapse. The data in the literature are inconsistent [18, 22]. Identification of statistically significant risk factors for CNS relapse was presented in two previously published studies. The risk factors for CNS recurrence reported by the previous research were lumbar puncture at diagnosis and LDH in one cohort [22] or age, lumbar puncture and MYCN amplification in another cohort [12]. In our group, disseminated disease in infants with MYCN amplification was the risk factor for the early CNS relapse (isolated CNS relapse was found in 25% of all MYCN amplified infants). HDC alone, probably, does not prevent CNS relapse [27]. In the presented data, occurrence of isolated CNS relapses is slightly higher in intensively treated patients with the employment of HDC, but the difference is not statistically significant.

Treatment of this kind of relapse is not established. Chemotherapy has been previously reported to be effective in single cases of patients with CNS NBL relapses, especially when there was the possibility of removing the tumour [24, 28]. With the employment of new treatment modalities, such as a protocol employing intrathecal radiolabelled antibodies [29], this kind of relapse is potentially curable. Moreover, therapy with intrathecal radiolabelled antibodies allows for the reduction of the radiotherapy dosage, which is extremally important in the context of expected toxicities, especially in small children [30].

Results of treatment with chemotherapy and radiotherapy are poor. One of the factors that may influence the prognosis is time to relapse. In our group, only in 3 patients the time to relapse was over 20 months, including 2 patients who survived. In the SIOPEN study, the median time to CNS recurrence from diagnosis was also shorter in comparison to relapses at other sites (*p* = 0.05) [26]. The longer time to relapse seems to be a better prognostic factor, also for the whole group of NBL patients, independently on the site of relapse [31].

In all reported patients, the parenchymal or leptomeningeal involvement were isolated settings. This supports the theory that CNS metastases are mainly blood-borne [22, 29] and that they may be related to genetic alterations of NBL cells during intensive therapy [12, 32, 33]. Specific genomic lesions, like 18q22.1 gain, may predispose to CNS metastases [34]. These lesions are recurrently acquired during metastatic progression. The TERT gene (5p), associated with telomere maintenance and poor prognosis in NBL [35], is one of the candidate genes associated with CNS involvement [34]. Patients with CNS relapse also have a different specific pattern of microRNA expression, with downregulation of miR-29a as a potential biomarker, potentially playing a role in CNS progression [36]. NBL cells penetrating into the CNS in a haematogenous way may keep their proliferative potential and may be a source of metastases [19, 29, 37]. In some studies, increased incidence of CNS metastases was observed after chemotherapy intensification [22, 28]. Our results also suggest that the natural history of the disease may be changed by intensive treatment. The reason for this may be the disruption of the blood–brain barrier, especially in very young children.

The symptoms of relapse are as described in previous reports. However, it’s important to underline that symptoms are not always present, especially in young children. In our setting, we found patients who either had no clinical symptoms and relapse was found on routine imaging (two patients) or who had severe neurological symptoms of bleeding and invagination was the first clinical presentation (four patients). Intraparenchymal haematoma occurring prior to radiologically detectable CNS metastases are rarely described in the literature [38].

Disease control in other tissues may be improved in the case of carrying out HDC followed by ASCR and immunotherapy [39]. It may decrease the incidence of disseminated relapses and increase the number of isolated relapses, including CNS relapses [25]. The diagnosis of isolated CNS relapse may be a more difficult challenge than disseminated relapses involving the CNS. Routine CNS examination must be taken into consideration at the scheduled time of disease assessment, even without having clear symptoms of CNS involvement reported. Also, whenever neurological symptoms occur during therapy, the relapse should be excluded. MIBG examinations performed at routine time points may not reveal metastases. Evaluation of CNS relapses in MIBG scintigraphy may be difficult; the CNS lesions may be interpreted as skull lesions and leptomeningeal infiltration may be not seen. Moreover, it was described in the literature that MIBG scintigraphy may be negative in confirmed relapses, even in nodular lesions [12]. Although NBL cells are found in histopathology at the time of intracranial bleeding, there may be no radiological symptoms of tumours present during radiological examinations, especially if CT/MRI are carried out without contrast.

This is a retrospective multicentre study, so the authors cannot exclude that some cases of CNS relapses might have been not reported or diagnosed, which is a limitation of the study. However, in all centres the routine imaging of CNS was performed at the most important time points of response evaluation, which let diagnose also asymptomatic relapses.

## Conclusions

CNS as a “pharmacological sanctuary” may be a new challenge for relapse diagnosis and treatment, especially with immunotherapy, significantly decreasing the risk of relapses in other organs. Brain evaluation should be recommended for all patients at the time of diagnosis, the time of relapse and at scheduled time points crucial for further therapeutic decisions. This should be also always performed when neurological symptoms occur before they can be interpreted as therapy toxicities, especially during immunotherapy [40]. Stage 4 infants with MYCN amplification must be taken into special consideration, as isolated CNS relapse may occur earlier during the course of therapy in this group of patients and have unspecific clinical presentation.

## Data Availability

The datasets generated and/or analysed during the current study are not publicly available due to General Data Protection Regulations but are available from the corresponding author on reasonable request.

## Abbreviations

CNS: Central nervous system
NBL: Neuroblastoma
PPSTSG: Polish Paediatric Solid Tumours Study Group
HDC: High dose chemotherapy
ASCR: Autologous stem cell transplantation
HR-NBL-1/SIOPEN: High Risk Neuroblastoma Study 1.0 of SIOP-Europe
SIOPEN: International Society of Paediatric Oncology European Neuroblastoma Research Network
Gy: Grey
CT: Computed tomography
MRI: Magnetic resonance
Dx: Diagnosis
BM: Bone marrow
LN: Lymph nodes
UH: Unfavourable histopathology
FH: Favourable histopathology
Y: Years
m: Months
M: Male
F: Female
RTX: Radiotherapy
CR: Complete remission
PR: Partial remission
MRD: Minimal residual disease
MIBG: Meta-iodobenzylguanidine
